# The cultural evolution of teaching

**DOI:** 10.1017/ehs.2023.14

**Published:** 2023-05-12

**Authors:** Eva Brandl, Ruth Mace, Cecilia Heyes

**Affiliations:** 1 Lise Meitner Research Group BirthRites, Department of Human Behavior, Ecology and Culture, Max Planck Institute for Evolutionary Anthropology, Leipzig 04103, Germany; 2 Department of Anthropology, University College London, London WC1E 6BT, UK; 3 All Souls College and Department of Experimental Psychology, University of Oxford, Oxford OX1 4AL, UK

**Keywords:** Cultural evolution, social learning, teaching, cognitive gadgets

## Abstract

Teaching is an important process of cultural transmission. Some have argued that human teaching is a cognitive instinct – a form of ‘natural cognition’ centred on mindreading, shaped by genetic evolution for the education of juveniles, and with a normative developmental trajectory driven by the unfolding of a genetically inherited predisposition to teach. Here, we argue instead that human teaching is a culturally evolved trait that exhibits characteristics of a cognitive gadget. Children learn to teach by participating in teaching interactions with socialising agents, which shape their own teaching practices. This process hijacks psychological mechanisms involved in prosociality and a range of domain-general cognitive abilities, such as reinforcement learning and executive function, but not a suite of cognitive adaptations specifically for teaching. Four lines of evidence converge on this hypothesis. The first, based on psychological experiments in industrialised societies, indicates that domain-general cognitive processes are important for teaching. The second and third lines, based on naturalistic and experimental research in small-scale societies, indicate marked cross-cultural variation in mature teaching practice and in the ontogeny of teaching among children. The fourth line indicates that teaching has been subject to cumulative cultural evolution, i.e. the gradual accumulation of functional changes across generations.

## Graphical abstract

**Figure:**
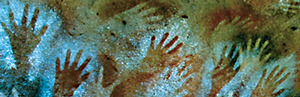

**Social media summary:** Teaching is an important means by which we transmit culture. We argue that teaching is itself thoroughly cultural.

## 1. Introduction

Where does teaching come from? What are the evolutionary and developmental origins of the capacity to teach? Researchers working on this question – in anthropology, biology, education, psychology and sociology – define teaching in two ways. The ‘functional’ definition, favoured by evolutionary biologists, casts teaching as a cooperative social interaction in which a knowledgeable individual modifies their behaviour in a way that has the effect of helping a naive individual to learn (Caro & Hauser, 1992). In this view, teaching occurs when a knowledgeable individual ‘modifies its behavior only in the presence of a naïve observer, […] at some cost or at least without obtaining an immediate benefit for itself’ (Caro & Hauser, 1992: 153), and as a result, the learner acquires the relevant piece of information more easily than they would have otherwise (Caro & Hauser, 1992). In line with their focus on the mind, many psychologists employ a ‘mentalistic’ definition of teaching, which reserves the term for cases in which a knowledgeable individual modifies their behaviour with the intention of helping another agent to learn (Frye & Ziv, 2005; Strauss et al., 2015). While the functional definition targets the adaptive function of teaching, the mentalistic one also considers cognitive mechanisms.

The behaviours covered by the two definitions overlap to a large extent. These include different types of teaching, for example: ‘social tolerance’ (a teacher allows a learner to closely observe their activities, tolerating physical proximity and intrusive behaviours such as touching), ‘opportunity provisioning’ (a teacher modifies an activity in a way that makes it less difficult or less dangerous for the learner), ‘stimulus enhancement’ (a teachers draws a learner's attention to an object or activity), ‘evaluative feedback’ (a teacher responds positively to good performance and negatively to mistakes from the learner), and ‘direct active teaching’ (a teacher uses verbal explanations and/or demonstrations) (Kline, 2015, 2017). These types of teaching have different effects: social tolerance allows the learner to scrutinise the teacher's behaviour; it can be distinguished from non-teaching forms of tolerance by the teacher's sensitivity to knowledge gains for the learner, showing more tolerance when learning benefits are greater (Kline, 2015, 2017). Opportunity provisioning allows learners to participate in a task that would be too dangerous or difficult to perform without help (Kline, 2015, 2017). Stimulus enhancement introduces the learner to objects and tasks to which they would not otherwise have paid attention (Kline, 2015, 2017). Evaluative feedback provides them with information about the outcomes of their actions and/or makes these outcomes more salient (Kline, 2015, 2017). Finally, direct active teaching provides information that cannot be inferred from observation alone (Kline, 2015, 2017). The main difference between the two definitions in terms of the behaviours covered is that, unlike the functional definition (which requires knowledge gain), the mentalistic one also incorporates failed teaching. This refers to cases in which a knowledgeable individual tries to help a novice acquire a skill but the learner fails to pick up the information (Frye & Ziv, 2005; Strauss et al., 2015).

Biologists using the functional definition have documented teaching in a range of non-human animals, including meerkats (*Suricata suricatta*) (Thornton & McAuliffe, 2006), tandem-running ants (*Temnothorax albipennis*) (Richardson & Franks, 2006), pied babblers (*Turdoides bicolor*) (Raihani & Ridley, 2008), superb fairywrens *(Malurus cyaneus*) (Kleindorfer et al., 2014) and golden lion tamarins (*Leontopithecus rosalia*) (Rapaport, 2011; Troisi et al., 2018). There are also reports of teaching in cheetahs (*Acinonyx jubatus*), domestic cats (*Felis catus*), domestic fowl (*Gallus gallus domesticus*), honeybees (*Apis mellifera*) (see Hoppitt et al., 2008), and controversially, among chimpanzees (*Pan troglodytes*) (Boesch, 1991; Musgrave et al., 2016, 2020). In contrast, humans are the only species that is widely believed to be capable of intentional teaching. Indeed, some researchers regard intentional teaching as a distinctively human trait – a characteristic that is present in all human societies, absent in all non-human animals, and crucial to our success as a species (Strauss & Ziv, 2012).

Although they are divided on many issues, there is a consensus among supporters of both the functional and mentalistic approaches that teaching is a ‘cognitive instinct’ (Pinker, 1995). They argue or assume that teaching depends on psychological processes that have been shaped at the population level by natural selection acting on genetic variants (henceforward ‘genetic evolution’), and at the individual level by a specific, genetically inherited predisposition to teach. This is implied in discussions among evolutionary biologists about the conditions in which teaching can evolve (Caro & Hauser, 1992; Hoppitt et al., 2008; Thornton & Raihani, 2008; Fogarty et al., 2011; Kline, 2015), and evident in the view advanced by psychologists that teaching is a form of ‘natural cognition’ (Strauss et al., 2002; Strauss & Ziv, 2012) or a manifestation of a genetically inherited propensity to engage in ‘shared intentionality’ (Burkart et al., 2009; Pradhan et al., 2012; Tomasello & Rakoczy, 2003; see also Ashley & Tomasello, 1998).

In this *Perspective* we offer an alternative evolutionary-developmental account suggesting that human teaching is a ‘cognitive gadget’ (Heyes, 2018, 2019a). We argue that the distinctively human features of teaching, some highlighted by the mentalistic definition, depend on psychological processes that have been shaped by natural selection acting on socially inherited variants (henceforward ‘cultural evolution’), which entails that teaching is transmitted between individuals through cultural learning. In other words, children learn to teach by interacting with other members of their social group. This learning is made possible by a range of genetically inherited resources that humans share with other animals, but it is not guided by a specific instinct to teach or to share intentionality. While our account addresses cognitive mechanisms, we thus take a different, more open-ended view of the cognitive machinery that distinguishes human teaching from that of other species. We therefore use the term ‘teaching’ in the functional sense, to subsume all the behaviours covered under that definition.

In Section 2 we review arguments and evidence supporting the cognitive instinct view of human teaching, focusing on the work of developmental psychologists and education researchers who have made the case most explicitly and persuasively. In Section 3 we develop the cognitive gadget account, and review evidence that supports this view. This evidence comes from experimental research on teaching in industrialised societies, from field work indicating cross-cultural diversity in the mature practice and childhood development of teaching, and from the ethnographic record pointing to the cumulative cultural evolution of teaching practices. In the final section we compare teaching with other cognitive gadgets and identify priorities for future research.

## 2. Human teaching as a cognitive instinct

The most forceful and eloquent proponents of the cognitive instinct view of human teaching are developmental psychologists committed to the mentalistic definition of teaching. They argue that, to be worthy of the name, teaching must depend on mindreading, also known as ‘mentalising’ and ‘theory of mind’, or the ability to mentally represent the thoughts, feelings, and perceptions of other agents (Strauss et al., 2002; Strauss & Ziv, 2012). This ability allows the teacher to take the learner's perspective, and therefore to understand that the learner knows less than themselves or might hold misconceptions about the skill that is being taught (Strauss & Ziv, 2012).

This group of psychologists, Strauss and colleagues, have argued that human teaching is a form of ‘natural cognition’ (Strauss et al., 2002) or ‘natural cognitive ability’ (Strauss & Ziv, 2012). They do not use the term ‘cognitive instinct’ or refer explicitly to evolution. However, when defining natural cognition, they apply the criteria developed by evolutionary psychologists Cosmides and Tooby (n.d.) to characterise what we, following Pinker (1995), call a cognitive instinct – a psychological process or set of processes that has been shaped at the population level by genetic evolution, and that develops in individuals via the unfolding of a specific genetic predisposition.

Using the template provided by Cosmides and Tooby (n.d.), Strauss and colleagues argue that human teaching has seven features indicative of a natural cognitive ability or cognitive instinct. It is (1) ‘complexly structured for solving a specific type of adaptive problem’, (2) distinct from more general abilities to process information, (3) applied without conscious awareness of the underlying logic, (4) species-unique (absent in non-human animals) and (5) species-typical (present in all human societies). Human teaching also (6) shows a normative developmental trajectory (typical development occurs in the same sequence across societies) and (7) develops without ‘formal instruction’ (Strauss & Ziv, 2012: 187). Strauss and colleagues focus on (6), saying relatively little about (1)–(5) and (7). Following their lead, we will not dwell on (1)–(4) because these criteria do not have the potential to support the instinct view over the gadget view, or vice versa. There is no reason to suppose that, in general, a phenotype shaped by genetic evolution will be more or less adaptive, complex, domain-specific, automatic or species-unique than a phenotype shaped by cultural evolution. For example, literacy – the capacity to read and write – is a culturally evolved cognitive ability that is high on all these dimensions (Heyes, 2012). We agree that teaching (functionally defined), is ubiquitous in human societies (5), but will argue that the substantial cross-cultural variation in teaching practices points to cultural evolutionary origins. We also agree that teaching may develop without direct instruction (7) but will argue that this does not negate a cultural evolutionary account; there are many processes of cultural transmission in addition to direct instruction. However, first we survey evidence for (6), suggesting that teaching shows a normative developmental trajectory.

Experimental studies of children in industrialised societies with high levels of formal education (such as France, Israel, the United States, Singapore, South Korea and Hong Kong) indicate a normative developmental sequence. At the earliest stage, infants perform a kind of proto-teaching. Eighteen- and 24-month-olds spontaneously intervene to correct an adult who is about to make a mistake by pointing out the correct location of an object the adult is looking for, suggesting that infants anticipate mistakes and intervene proactively (Knudsen & Liszkowski, 2012a, b). Later, when teaching peers, 3-year-olds have been found to predominantly rely on non-verbal teaching strategies, such as demonstrating skills or physically intervening in the learner's actions, combined with simple forms of verbal communication, such as short commands (Strauss et al., 2002; Ziv et al., 2016; Ronfard & Corriveau, 2016). In contrast, 5- and 6-year-olds rely more on verbal communication, especially abstract communication and elaborative teaching using verbal explanations (Strauss et al., 2002; Ziv et al., 2016; Ronfard & Corriveau, 2016; Ye et al., 2021). From ages 5 and 6, children are also better able to combine words and gestures to communicate with the learner, pay more attention to the learner's level of understanding and are more flexible in tailoring instruction to the learner's needs, goals and competence (Strauss et al., 2002; Davis-Unger & Carlson, 2008; Bensalah, 2011; Ziv et al., 2016; Gweon et al., 2018; Gweon & Schulz, 2019; Bridgers et al., 2020; Ye et al., 2021). These shifts are correlated with children's developing understanding of false belief (children's awareness that other people can hold misconceptions; Strauss et al., 2002; Davis-Unger & Carlson, 2008; Ziv et al., 2016; Ye et al., 2021).

These behavioural shifts are accompanied by changes in children's reflections about teaching. When reflecting about how they taught a peer, 3-year-olds have been found to focus on the content (what they taught), whereas 4- and 5-year-olds also show awareness of the transmission process (how they communicated with their peer; Strauss et al., 2002; Davis-Unger & Carlson, 2008). When reflecting about whether a learner has mastered a game, 3-year-olds tend to treat the fact that they taught as evidence that their peer has learned the skill (‘I know that he learnt it because I taught him!’) (Strauss et al., 2002; Davis-Unger & Carlson, 2008). In contrast, 4- and 5-year-olds are more attentive to the learner's behaviour (‘I know that he learnt it because he played well’) and can infer the learner's knowledge from mistakes they have made (Strauss et al., 2002; Davis-Unger & Carlson, 2008; Ronfard & Corriveau, 2016). Moreover, unlike 3-year-olds, 5-year-olds have been found to recognise teaching as an intentional activity; they can distinguish between intentional teaching (where a knowledgeable individual intends to transmit information to a naive learner) and imitation (where a naive learner merely copies a knowledgeable individual) (Ziv et al., 2008; Jeong & Frye, 2018). By age 6–7, children can also articulate process-based definitions of teaching (Sobel & Letourneau, 2016). Finally, 3- and 4-year-olds have been found to recognise that to transmit information, knowledgeable individuals should teach naive ones (Ziv & Frye, 2004; Bensalah et al., 2012; Ziv et al., 2016). However, only by age 5–6 do children start to recognise that teachers also act on beliefs about their own and the learner's knowledge level – and that these beliefs can be false (Ziv & Frye, 2004; Bensalah et al., 2012; Ziv et al., 2016; Wang et al., 2017; Bass et al., 2021).

In summary, the evidence supporting the cognitive instinct (or natural cognition) account of human teaching comes primarily from carefully conducted studies of children in industrialised societies. These studies suggest a typical developmental sequence in which children's dominant strategy shifts from demonstrations and short commands at age 3 to abstract verbal communication at age 5, from reflecting on what they taught to reflecting on how they taught, and from treating teaching as evidence of learning to treating the learner's behaviour as evidence of learning. From about 3–4 years old, children in industrialised societies believe that knowledgeable individuals should teach naive ones.

## 3. Human teaching as a cognitive gadget

### 3.1. The gadget hypothesis

The alternative, cognitive gadget view suggests that cultural evolution, rather than genetic evolution, has played the dominant role in the emergence of human teaching. It proposes that the cognitive capacity to teach is assembled from ‘old parts’ during childhood through cultural learning – by observing and interacting with older children and adults as these experts exercise their more advanced capacity for teaching. The development of cultural learning is facilitated by a ‘genetic starter kit’, a set of genetic adaptations specific to the hominin line that make us peculiarly receptive to information from other agents, including enhanced social tolerance, attention and motivation, and expanded capacities for associative learning and executive controls. However, these genetically inherited resources are not specific to the development of teaching. Rather than being a programme for the development of teaching, of the kind implied by the instinct view, the starter kit functions to promote all kinds of social and cultural learning – for example, learning about foraging, tool use and language, as well as learning to teach (Heyes, 2018).

The cognitive ‘parts’ assembled by cultural learning into a capacity for teaching include: perceptual and attentional processes that enable the teacher to spot errors, i.e. differences between naive and expert performance; learning processes that track the novice's progress toward expert performance and the effectiveness of the teacher's verbal and non-verbal interventions in advancing that progress; executive processes allowing the teacher to switch interventions to advance the learner's progress, and to inhibit the impulse to take over; and mindreading, through which the teacher represents learners, not only in terms of what they do, but also in terms of what they know. Many of these parts are both phylogenetically and ontogenetically ‘old’; they are evolutionarily ancient and come online early in human development, long before children begin to teach. For example, reinforcement learning of the kind that tracks a novice's progress (Apps et al., 2005) is present in a broad range of vertebrate and invertebrate species (MacPhail, 1982; Shettleworth, 2010) and early in human infancy (Siqueland & DeLucia, 1969). Even mindreading, the most human-specific and late-developing part, is ‘old’ in the sense of having evolved – genetically or culturally (Heyes & Frith, 2014) – to fulfil a wide range of social functions. It is sometimes *used* for teaching, but it was not *made* for teaching. Even supporters of the instinct view, who see mindreading as the defining feature of teaching, do not argue that mindreading evolved specifically for teaching (Strauss & Ziv, 2012).

The gadget perspective does not cast mindreading as the central or defining feature of teaching. Instead, motivated by research indicating cultural diversity in the mature practice and development of teaching (see below), we suggest that, during ontogeny, humans assemble different cognitive packages for teaching depending on their socio-cultural environment. The packages contain components of roughly the same kinds – perception, attention, learning, executive processes and mindreading – but the relative importance of these components, and the way they are configured vary widely depending on local ecology, what needs to be taught within a society, and cross-cultural contact, the extent to which a society has been exposed to the teaching gadgets of other cultures.

Social anthropologists have long argued that teaching practices associated with formal education, such as direct active teaching and frontal instruction, are culturally transmitted and can diffuse across cultures (Lancy, 2015; LeVine et al., 2012). The gadget hypothesis develops this view by incorporating all forms of teaching and extending the focus from behaviour to cognition, proposing that there is cultural transmission, not only of ways of acting, but also of ways of thinking. It suggests that teaching hijacks cognitive mechanisms involved in bonding, coordination and communication more generally along with domain-general abilities such as reinforcement learning and executive function. The kind of teaching children receive shapes their teaching-related behaviour and cognition in a synthetic, plastic ontogenetic process.

The gadget hypothesis does not merely acknowledge that the development of teaching is modulated by cultural factors. That is true of the vast majority of, if not all, human traits. Rather, the gadget hypothesis suggests that, without exposure to teaching by others in their society, children would not learn to teach at all, or would teach in the same simple ways observed in other animals. These sadly deprived children would have the cognitive resources provided by the genetic starter kit and could use them to learn by observing others and by direct engagement with the inanimate world. However, without models of teaching behaviour, they would develop, at best, a rudimentary capacity to teach others – inferior to that observed in any contemporary human society.

Children probably learn to teach in the first instance through experiencing communicative acts that are embedded in everyday life. Depending on the cultural context, this may involve eye contact, pointing and infant-directed speech, or situations in which caregivers orient the child's body towards interactions between other people. Children's attentional bias towards other people and their prosocial emotions motivate them to join these interactions. As a result, children are increasingly exposed to teaching events. In many small-scale societies, this occurs when caregivers allow them to interact with objects they are using, give them commands or assign simple chores to them. The tasks that children participate in get more complex with age, which necessitates more costly forms of teaching such as detailed demonstrations. As children experience this process, they start to emulate and imitate what they see and hear, using abstract communication, demonstrations or task assignment when they interact with their peers, depending on what they have been exposed to. Children might receive situational feedback that helps them refine their own teaching and adjust to the abilities of others – for example, when they try to engage a younger sibling in difficult activities such as nut-cracking but are told that their sibling is too small to hold the hammer. As they get older, they eventually become receptive to the ethno-theories of teaching, knowledge and authority endorsed in their particular cultural environment (rather than developing a discrete, universal notion of teaching), although they probably do not fully grasp these until adolescence or even adulthood. Teaching thus relies on a genetically inherited starter kit made up of prosocial psychological mechanisms but also needs regular teaching interactions with caregivers and peers to get off the ground. This process probably involves a combination of multiple different mechanisms of cultural transmission (such as observation, imitation and teaching by evaluative feedback); our account does not imply that teaching is primarily learnt through active instruction (i.e. that caregivers take children aside to instruct them in how to teach).

We now present four lines of evidence which together support the cultural evolutionary account of teaching. The first, based on psychological experiments in industrialised societies, indicates that domain-general cognitive processes – old parts – are important for teaching. The second and third lines, based on naturalistic and experimental research in small-scale societies, indicate marked cross-cultural variation in mature teaching practice, and in the ontogeny of teaching among children. The fourth line indicates that teaching has been subject to cumulative cultural evolution, i.e. the gradual accumulation of functional changes across generations.

These lines of evidence are convergent. By itself, the evidence of cross-cultural variation (second and third lines) could be taken to show only that the development of a cognitive instinct for teaching is sensitive to a person's cultural context. However, when evidence of cultural variation is combined with evidence that teaching depends on a range of domain-general cognitive processes (first line) and has been subject to cumulative cultural evolution (fourth line), the gadget hypothesis – postulating a minimal genetic starter kit and a dominant role for cultural selection – becomes more plausible than the nativist alternative.

### 3.2. Teaching from old parts

The gadget account suggests that a mature cognitive capacity to teach is a culturally variable configuration of domain-general processes, in which mindreading does not consistently play a dominant role. In contrast, the instinct view places mindreading, the most socially specific component of the configuration, at centre stage. It defines teaching in relation to the mental capacity to represent a knowledge gap between the teacher and the learner (Strauss et al., 2002; Ziv & Frye, 2004).

Recent studies of children in industrialised societies suggest that mindreading is less important for teaching than the instinct view suggests. For example, when 3–6-year-olds in the US were told that one puppet ‘knew how to play the game’ and another ‘had never seen the game before’, they answered correctly when asked which puppet was knowledgeable, but they were no more likely to teach the puppet with whom they had a knowledge gap, than the puppet that already ‘knew how to play the game’ (Corriveau et al., 2018; Ronfard et al., 2015). In a more extreme case, 3–6-year-old children in the US positively preferred to impart information to a more knowledgeable individual than to a less knowledgeable individual, even when the former had stated plainly that they already knew the information to be taught (Kim et al., 2016).

Other research of yet broader significance suggests that executive functions may be more important than mindreading for the development of teaching in industrialised societies (Davis-Unger & Carlson, 2008). Executive functions – including inhibition, attention, working memory and error detection – are domain-general processes that monitor and control all kinds of thought and action, from manual skills such as reaching and grasping, through emotion regulation, to higher cognitive functions such as financial reasoning. Davis-Unger and Carlson (2008) used a range of standard tests to assess mindreading and executive function development in a sample of US children between 3.5 and 5.5 years of age. They then taught each child to play a ‘flower game’, a board game developed by Strauss et al. (2002), and asked them to teach an adult to play the game. Both mindreading (three tasks) and executive function (five tasks) composite scores predicted the children's teaching skill (e.g. time spent teaching, the range of strategies used, the number of rules taught and the number of errors detected), indicating that both contributed to teaching efficacy. Crucially, however, executive function was a better predictor than mindreading of the children's teaching skills. This suggests that US children depend more on domain-general executive processes than on mindreading when they are teaching. It also raises the possibility that other studies of Western children systematically underestimate the contribution of executive processes. For example, Ziv et al. (2016) found that performance on standard tests of mindreading predicted teaching skill in 3–5-year-old Israeli children and interpreted this as evidence that mindreading is the crucial component of teaching. However, performance on tests of mindreading depends in part on executive functions, and Ziv et al. did not independently assess executive function. Therefore, it is possible that the teaching skill of the children in this study depended as much or more on executive function as on mindreading.

Turning from executive function to reinforcement leaning, a yet older and more domain-general process, brain imaging of Western adults indicates that reinforcement learning plays a significant role in teaching. This kind of associative learning, which proceeds via the detection and correction of prediction errors (the difference between an action outcome and a goal state; Rescorla & Wagner, 1972), is a very ‘old part’. It contributes to learning all motor skills, is present in a wide range of species and comes online early in human infancy (MacPhail, 1982; Siqueland & DeLucia, 1969). Apps et al. (2015) taught adults in the UK to press one of four keys when they saw each of four colours, and then asked them to teach this skill to another person by indicating whether each of the pupil's actions was right or wrong. They found that activity in the teacher's anterior cingulate cortex when they saw the pupil's response correlated with the prediction error of that response. Given that the anterior cingulate cortex signals prediction errors in ‘standard’ reinforcement learning, when an agent is learning from the outcomes of their own actions, this result suggests that ancient mechanisms of associative learning are recruited for teaching.

Building on the theory of ‘natural pedagogy’ (Csibra & Gergely, 2006, 2009, 2011), some psychologists have proposed that, rather than co-opting ancient mechanisms of reinforcement learning, human teaching is an inherently communicative process that depends on mindreading by both teacher and pupil (Ho et al., 2017, 2019; see also Heyes, 2019a). The theory of natural pedagogy suggests that human infants are born with a package of specific genetically inherited dispositions preparing them to be taught. These include a genetically inherited sensitivity to ostensive signals such as infant-directed speech and eye contact, a propensity to engage in turn-taking (contingent reactivity), and a tendency to follow eye movements (Csibra & Gergely, 2009, 2011). In contrast with the cognitive gadget account, which suggests that infants are receptive to teaching by virtue of domain-general prosocial adaptations, the theory of natural pedagogy claims that human infants are born to ‘expect to receive ostensive-referential communication from adults’ and to ‘expect to learn something generalisable in ostensive-referential contexts’ (Csibra & Gergely, 2009: 151). However, close examination of the evidence for natural pedagogy suggests that it is not sound (Heyes, 2016a). For example, human infants are attentive to eye contact (Legare & Harris, 2016), but they do not consistently prefer direct eye contact to averted gaze (Vecera & Johnson, 1995; Farroni et al., 2006), and attention to eye contact is found in many species that do not teach (Emery, 2000). Similarly, a taste for contingent reactivity is found in many social species, including rats (Werner & Latane, 1974) and precocial birds (Harshaw & Lickliter, 2007; Harshaw et al., 2008), suggesting that it not a ‘new part’ built for teaching but an ‘old part’ built for social bonding (Heyes, 2016a).

Arguably, there is one ‘new part’ that is specific to human teaching: conceptual communication through language. Some have argued that the ability to communicate concepts may have been particularly beneficial for complex technical skills such as the construction of Acheulian hand axes (Chazan 2012; Gärdenfors & Högberg 2017). Conceptual teaching can overcome teleological opacity (by making the goals of knowledgeable individuals more explicit) and causal opacity (by illuminating the causal relationships between different aspects of a task) (Hernik & Gergely 2015). Some have used this to advance the position that, in humans, teaching co-evolved with complex tool use and language (Laland 2017; Lombao et al., 2017; van Schaik et al., 2019; see also Battro 2010). However, experiments suggest that the link between verbal teaching and stone tools may in fact be rather tenuous. While some have found that these skills are more efficiently transmitted through verbal communication (Morgan et al*.,* 2015), others observed no such benefit (Ohnuma et al., 1997; Putt et al., 2014; Cataldo et al., 2018). Accordingly, we believe that the cognitive abilities utilised in conceptual communication and language were probably selected because they are beneficial for solving a wide range of coordination problems, not specifically for teaching.

Studies conducted in industrialised societies have found that children and adults often depend on representation of their pupils’ mental states when teaching. This is established by the work of Strauss and colleagues reviewed in Section 2 and by Ho et al. (2019). However, consistent with the gadget account of teaching, the research sampled in this section indicates that mindreading is less important, and ‘old parts’ – such as executive functions and reinforcement learning – are more important, than the cognitive instinct view implies. Furthermore, ‘new parts’ that are specific to humans – such as language – probably did not evolve specifically for teaching.

### 3.3. Cultural diversity in teaching

Children must work ‘under adult guidance or in collaboration with more capable peers’ (Vygotsky, 1978: 86) to develop abilities they cannot yet perform independently (the zone of proximal development, see Vygotsky, 1978). Teaching therefore occurs in all human cultures (Kline et al., 2013; Kline, 2015). However, unlike non-human animals – where teaching is often limited to key aspects of food acquisition, with a single target behaviour taught in each species (Premack, 2007) – humans show numerous cross-cultural differences in *what* is taught and *how* the teaching is done.

In societies with formal education systems, children spend much of their time supervised and taught by professional teachers who are not related to them. Formal education emphasises frontal teaching, direct instruction and abstract verbal explanations (Scribner & Cole, 1973; Legare, 2017, 2019). Additionally, middle-class parents in Western countries often practice school-like interactions through intensive verbal communication with their children, often teaching skills (like walking) that do not need to be taught (Rogoff, 2003; LeVine et al., 1994; Morelli et al., 2018; Lancy, 2015; Legare, 2017, 2019). However, in some hunter–gatherer and mixed subsistence societies, adults rarely talk to young children. For example, among Tsimane forager–horticulturalists in lowland Bolivia, toddlers under 4 years of age receive less than a minute of ‘talking time’ per daylight hour (Cristia et al., 2019). In many small-scale societies, much learning is done by observation, imitation and pretend pay, which do not involve direct communication with an adult caregiver (Scribner & Cole, 1973; Gaskins & Paradise, 2010; Legare, 2017, 2019). This is evident in African farmers and foragers (Hewlett et al., 2011; Boyette, 2016; Boyette & Hewlett, 2017; Lew-Levy & Boyette, 2018).

When they do teach, caregivers often employ less costly forms of teaching, for example by facilitating ‘learning by doing’: children learn skills as they participate in everyday activities, observing others and contributing as opportunities arise (Scribner & Cole, 1973; Rogoff, 2003; Paradise & Rogoff, 2009; Legare, 2017, 2019). This approach emphasises ‘[l]earning “by osmosis”, picking up values, skills, and mannerisms in an incidental fashion through close involvement with a socializing agent’ (Rogoff, 2003: 323). Caregivers primarily use speech to support the activities they are engaging their children in, for example by giving commands, instead of giving advance verbal instruction (Paradise & Rogoff, 2009; Scribner & Cole, 1973; Rogoff, 2003). They also emphasise forms of non-verbal communication such as touch and gesture (Legare, 2017, 2019). This has been observed among rural farming populations such as the Gusii in Kenya, where mothers usually speak to young children in short commands (LeVine et al., 1994). This preference is also evident in experimental studies. For example, in an experiment where caregivers taught a game to their child, US caregivers focused on direct active teaching and took on a leading role in interactions with the child (for example by guiding and praising them) (Clegg et al., 2021). In contrast, ni-Vanuatu horticulturalists from the South Pacific relied more on shared interaction styles (dividing the task between the caregiver and the child), reflecting their expectation that children should learn from collaboration and observation (Clegg et al., 2021).

Subtle forms of teaching also involve task assignment, where children are told to complete a simple chore (such as fetching a tool) (Rogoff, 2003; Lew-Levy et al., 2019). In a review of the ethnographic literature on hunting in hunter–gatherers and mixed-subsistence societies, MacDonald (2007) found that children usually start to gain experience with hunting weapons at a young age as adults and older children provide them with toy weapons to play with. Real weapons are increasingly provided as they grow older (MacDonald, 2007). Adults let children accompany them on hunting trips and sometimes facilitate learning by focusing on easy prey and providing them with opportunities to make their first kills (MacDonald, 2007). Among the Chabu in Ethiopia, children's fathers show them how to butcher meat; during hunting trips, adults respond to questions, show them how to perform important skills, tease them about mistakes and correct them (Dira & Hewlett, 2016). They also provide them with carcasses for practice (Dira & Hewlett, 2016). BaYaka caregivers use pointing, eye contact and child-directed speech to direct a child or infant's attention and to familiarise them with tools through negative feedback, demonstrations and opportunity scaffolding (providing the infant with an object to explore) (Hewlett & Roulette, 2016; Boyette & Hewlett, 2017). When hunter–gatherers and mixed subsistence societies use costly forms of teaching such as abstract verbal communication, this is usually done to communicate opaque knowledge such as social norms (Salali et al., 2019) and complex skills such as spear hunting (Lew-Levy et al., 2022). However, in many cases explanations of plant and animal knowledge still occur alongside hands-on practice and opportunity scaffolding (MacDonald, 2007; Lew-Levy et al., 2022).

There are also cross-cultural differences in who does the teaching. Formal education systems are guided by adult teachers. However, while adults play a prominent role in complex tasks such as spear hunting (Lew-Levy et al., 2022), much hunter–gatherer teaching occurs between children. Hadza and BaYaka children spend much of their time in child-only play groups; as a result, they receive more teaching from other children than from adults (Lew-Levy et al., 2020). There are also differences in the importance of kin. For example, siblings are more prominent teachers among the Hadza than among the BaYaka, which may reflect different settlement patterns (BaYaka children have more opportunities to interact with people outside the nuclear family; Lew-Levy et al., 2020).

Cross-cultural variation in teaching practices may be driven by a range of factors such as exposure to formal education and differences in the kinds of skills children must learn, which are shaped by the subsistence system (Legare, 2017, 2019). These driving factors can be differentiated into ontogenetic and functional explanations, which complement each other (Tinbergen, 2005; Micheletti et al., 2022a, b). Functionally, hands-on teaching as practised by foragers and subsistence agriculturalists may be especially effective at imparting the practical skills children need to survive in those environments. In contrast, abstract verbal communication prepares children for formal schooling, which enables them to gain resources through employment in a commercial economy. Numeracy and literacy may require training in a dedicated space separate from everyday life, which lends itself to the professionalisation of teaching. These contrasting ecologies thus favour the emergence of different teaching practices; children end up adopting the type of teaching they were exposed to during ontogeny.

This may help us understand why people from small-scale societies sometimes adopt more ‘schoolish’ communication styles after they have been exposed to formal education. Traditionally, Quechua caregivers in Peru teach mostly through non-verbal demonstrations and Guatemalan Maya have emphasised egalitarian ways of communicating with children (Chavajay & Rogoff, 2002; Visscher, 2010). However, caregivers with more years of schooling use more verbal instruction and more hierarchical interaction styles, resembling practices they have encountered in school (Chavajay & Rogoff, 2002; Visscher, 2010). In an origami folding task, ni-Vanuatu caregivers with more formal schooling also used a greater variety of teaching strategies (Boyette et al., 2022). During ontogeny, these caregivers may have adopted forms of instruction they encountered in the classroom and now apply them to their own children.

Other cross-cultural differences may come down to broader cultural values that do not necessarily map onto function. According to Corriveau et al. (2018), Chinese teachers often expect students to recognise if they do not understand a lesson and ask for help accordingly. In contrast, US teachers are expected to monitor the students’ understanding and explain information in multiple different ways, anticipating possible difficulties (Corriveau et al., 2018). In the Central African Republic, verbal instruction is more common among Ngandu farmers than Aka hunter–gatherers, reflecting the more hierarchical values of Ngandu society (Boyette & Hewlett, 2017).

The evidence on teaching practices in adults suggests that there is a great deal of cross-cultural variation, especially between hunter–gatherers and subsistence societies on the one hand and industrialised societies with high levels of formal education on the other. It also suggests that exposure to formal schooling can shift teaching practices in small-scale societies, promoting the use of more verbal instruction and/or more diverse teaching strategies by caregivers. This is consistent with the idea that teaching strategies are culturally learned.

### 3.4. Cultural diversity in the development of teaching

We have seen that active facilitation of learning occurs in one form or another across cultures, although the way skills are taught differs between cultures. This supports a cultural evolutionary account of teaching, but it is also important to examine evidence that children adopt the teaching methods and communicative styles to which they have been exposed. If the ontogeny of teaching differs across cultures, and the input that children receive shapes how they themselves teach, the idea that teaching practices are culturally transmitted is strengthened.

Indeed, tentative findings, primarily from cross-cultural experimental research, suggest that the diverse cultural inputs that children receive, and the teaching interactions they are exposed to, shape children's own teaching and the way they think about the transmission of information. For example, children's early social experiences appear to shape their perspective-taking in teaching situations. A screen-based study in the Netherlands showed that 5-year-olds can adapt their teaching behaviour to the (perceived) age and competence of the learner, spending more time at relevant game locations when they believed that the learner was a toddler rather than a same-aged peer (Stolk et al., 2013). Intriguingly, children who had spent more time in nursery were better able to take the learner's age and ability into account (Stolk et al., 2013). This suggests that children learn to refine their teaching through regular social interactions with other children.

Additionally, cross-cultural differences in the way caregivers interact with and instruct children may shape the developmental trajectory of children's teaching (see Corriveau et al., 2018). When teaching a game to a naive peer, children from rural Vanuatu, where early participation in productive activities is common in everyday life, have been found to use a participatory approach to teaching that emphasised learning-by-doing and short commands up until age 8 (Brandl et al., in press). Abstract teaching with verbal explanations only became common from age 9 onwards (Brandl et al., in press). These patterns differ from trajectories identified in industrialised societies, where abstract verbal instruction becomes the dominant strategy by age 5 (see Section 2). However, the pattern from Vanuatu resembles observations from Maya children. By age 4, Maya children looking after their younger siblings start to initiate teaching, but 3–5-year-olds mostly use commands instead of explanations or verbal feedback (Maynard, 2002). The latter only increase in 6–7-year-olds and their use expands further in 8–11-year-olds (Maynard, 2002).

Cross-cultural differences in the conceptualisation of teaching may also shape how children reason about teaching. When reflecting about how they taught their peer, nearly half of the 4–11-year-old ni-Vanuatu children who took part in Brandl et al. (in press) focused on the content (what they taught); reflection about the transmission process and their communication strategies only increased from age 9 onwards. When reflecting about whether the learner mastered the game, most children at all ages treated the fact that they taught as evidence that learning occurred, with only a minority taking the learner's actual behaviour into account (Brandl et al., in press). Again, this departs from patterns observed in industrialised societies with high levels of formal education, where children tend to emphasise their communication strategies and the learner's behaviour by age 5 (see Section 2). This may reflect local ethno-theories of knowledge production. The latter treat knowledge as an external reality that people can possess and exchange, and that exists independently of internal mental processes or specific communicative acts (Lindstrom, 1990). This ethno-theory also posits that learners acquire knowledge not through personal reflection, but through osmosis from legitimate authorities (Lindstrom, 1990).

Different conceptualisations of teaching have also been found in Chinese samples. Unlike Western children, who tend to state that a teacher should preferentially teach a naive learner (as opposed to an already knowledgeable one), Chinese children state that the already knowledgeable learner should be taught, presumably to improve their existing skills (unpublished study discussed in Wang et al., 2017). This may be due to differences between Kantian and Confucian philosophy and their cultural downstream effects: while the former defines learning as the process of acquiring knowledge, the latter also views learning as the process of perfecting oneself (Wang et al., 2017), which has influenced Chinese folk models of learning (Li, 2002). On the other hand, a separate study found that, compared with Germans, Japanese children showed a stronger tendency to preferentially teach a naive learner as opposed to a knowledgeable one, which may reflect an emphasis on interdependence and tending to others’ needs in Japanese children's family environments (Kim et al., 2018).

While cross-cultural research on the ontogeny of teaching is still emerging, the work published so far suggests that there is cross-cultural variation in the ways children teach and reason about teaching. Differences have been found between industrialised and subsistence societies, but also between different industrialised societies with equally high levels of formal education but different value systems and folk models of learning and knowledge. This constitutes direct evidence that teaching is culturally learned.

### 3.5. Cumulative cultural evolution of teaching

We have reason to believe that teaching is culturally learnt not only because teaching practices are diverse and developmentally plastic, but also because they are subject to cumulative cultural evolution (where the functionality or efficacy of cultural traits improves across cultural ‘generations’). In other words, teaching practices are gradually refined across generations. This is aided by a process in which social groups deliberately organise cultural transmission in a dedicated social context that is separate from everyday life (Scribner & Cole, 1973). We might call this ‘intensive teaching’ (when it occurs in small-scale societies, Scribner and Cole (1973) call it ‘noninstitutional formal education’). In small-scale societies, intensive teaching is mostly used for ceremonial and spiritual expertise transmitted during initiation rituals. When it was time for male initiations in Malakula (an island in Vanuatu), the fathers of the boys planted yam gardens for their sons and appointed some trusted men to guide them through the rituals (Deacon & Wedgwood, 1934: 250). They constructed a ceremonial house where the boys went into seclusion before being circumcised (Deacon & Wedgwood, 1934: 250). They were then ‘taught how to make and play the panpipes’ (Deacon & Wedgwood, 1934: 253). Furthermore, ‘the novices are made to witness at night a number of remarkable performances, innumerable and ingenious “hoaxes”’ (Deacon & Wedgwood, 1934: 253). Men dressed up as ghosts and spirits and frightened the boys (Deacon & Wedgwood, 1934: 254). Afterwards, ‘however, the secret of the deception is revealed to them; they are taught exactly how the “hoax” was carried out, […] and they learn how to perform it themselves against the time when, as grown men, they will have to play their part in the incision ceremonies’ (Deacon & Wedgwood, 1934: 255).

Intense, high-arousal rituals such as this one emphasise the experiential dimension of rituals and thus episodic memory over semantic knowledge, but they are still vehicles of cultural transmission (Whitehouse, 1992). The music sessions and the reveals after the hoaxes fit the criteria for teaching, but by removing the boys from their family environment, these initiations also carve out a social sphere dedicated to experiencing a ritual which the boys will in turn transmit to the next generation. This practice requires active coordination between multiple teachers and learners at the same time. It further requires that the novices’ families plan their resource use ahead of time, which is evident in the planting of dedicated yam gardens.

In large-scale societies, these dedicated spaces are found in modern education systems, but also in workshops run by medieval craftsmen and scholarly institutions such as monasteries. In economies characterised by increasing professional specialisation, these spaces allowed people not only to accumulate specialist skills, but also to engage in highly specialised methods of instruction that were transmitted along with these skills. For example, in some societies students learned how to paint by drawing live models under the guidance of a master instructor, or they learned about poetry by repeating a teacher's recital in unison. In this way, students learned not only how to paint or recite poetry, but also how to teach painting and poetry, by interacting with expert teachers from their culture. This was only possible because resource transfers, brokered by wealthy benefactors and political leaders, freed them from subsistence production. As the professions diversified in increasingly market-based economies, some aspects of cultural transmission became themselves subject to professional specialisation, resulting in the installation of full-time educators. Once this was accomplished, expertise about teaching could itself accumulate, which we can see in the field of didactics – where teaching is taught through direct instruction at tertiary institutions of formal education. Teaching therefore not only contributes to cumulative cultural evolution in the sense that it improves the transmission of complex skills (Caldwell et al., 2017; Lucas et al., 2020); it is also subject to cumulative cultural evolution.

## 4. Directions for future research

The gadget hypothesis suggests that a priority for future research on teaching, in psychology and anthropology, is to establish the extent to which teaching is a culturally learned skill. The research reviewed in Section 3 suggests that cultural learning plays a role in the development of teaching, but more definitive evidence is needed. To get this evidence, we need to prepare the conceptual ground and do some empirical digging.

To prepare the ground, we need to be clear about what we are looking for. Supporters of the cognitive instinct view of human teaching say that teaching develops without ‘instruction’ (Strauss & Ziv, 2012: 187), but instruction is not necessary for cultural learning. Indeed, cultural learning – social learning of a kind that can support inheritance and therefore cultural selection – does not require teaching either in the mentalistic sense (a deliberate attempt to impart knowledge) or even in the functional sense (costly modification of behaviour that promotes development of another's skill; Caro & Hauser, 1992: 153). Cultural learning requires only that contact between two agents, A and B, causes B to acquire information from A (Heyes, 2019b). I can culturally learn how to use a tool simply by watching you using the tool. It may help if you modify your behaviour while I am watching, with or without the intention of helping me to learn, but I can learn plenty about what the tool can do, how it should be held, and how it should be moved, even when you are oblivious to my presence. Similarly, I could learn how to teach by watching you teach a third party, or as your pupil when the function or intention of your behaviour is to improve my tool use rather than to teach me how to teach. Consequently, processes of cultural transmission such as observation, imitation and evaluative feedback would suffice to bring about mature teaching in most cases, with direct instruction in teaching limited to highly specialised settings such as didactics courses at universities.

Therefore, when it comes to the empirical digging – laboratory and field studies in a range of societies, testing the instinct and gadget accounts against one another – we are looking for evidence that the teaching strategies to which children are exposed shape their teaching behaviour, and their developing conceptualisations of the teaching process. Accordingly, the ontogeny of teaching and to what extent it is influenced by cultural factors should be a priority in future work. A particularly promising focus is suggested by recent work indicating that, although older people are often tasked with imparting knowledge to the young (Gurven et al., 2020; Schniter et al., 2018), hunter–gatherer children are frequently taught by their peers (Lew-Levy et al., 2020).

Another priority for future research is to develop a more explicit account of the cognitive processes that underwrite teaching skill. At present, both the instinct and gadget accounts portray these processes as a cluster of components; they say very little about how the components relate to one another. In contrast with the instinct view, the gadget account suggests that the relative importance of the components (e.g. mindreading and executive functions) and the way they fit together vary over time and across human societies. This is a substantial proposal, but it leaves open the question of whether these components are assembled into an integrated system and, if so, whether the system properties vary across cultures.

Research on other cognitive gadgets indicates that both high and low, as well as intermediate, degrees of integration are possible. For example, research on imitation – the capacity to copy how parts of the body move relative to one another (e.g. when learning culture-specific facial expressions and dance movements) – suggests a high level of integration. Children culturally learn a large repertoire of sensorimotor associations, linking the sight and performance of different body movements; these act as gears enabling one kind of sequence learning, encoding observed body movements, to drive another kind, encoding enacted body movements (Catmur et al., 2009; Heyes, 2015). There is also evidence that mindreading depends on a highly integrated, culturally learned cognitive system (Heyes & Frith, 2014). We have not discussed that evidence in this article to avoid giving the false impression that the gadget account of teaching depends on the gadget account of mindreading. However, it is worth noting that, even if human teaching was universally dependent on mindreading, it would not necessarily follow that human teaching is a cognitive instinct. At the other extreme of the integration continuum, truly strategic social learning – social learning filtered by deliberation about which agents are likely to ‘know best’ – co-exists in the human cognitive system with older mechanisms of selective social learning. The older and culturally learned selective mechanisms largely operate in parallel (Heyes, 2016b, c).

The gadget hypothesis offers a new perspective on the relationship between teaching in humans and other animals. In contrast with the instinct account, the cultural evolutionary hypothesis acknowledges that mindreading can contribute to teaching, but it does not cast mindreading as *the* defining feature of teaching in general or of human teaching in particular. Therefore, while some have argued that human teaching is qualitatively different from teaching in non-human animals (see Rodriguez, 2013), the gadget view opens the possibility that, at the level of cognitive components, there is substantial continuity. Mindreading may be rare or absent in other species, but many animals have the more domain-general processes – including reinforcement learning and executive functions – from which human teaching is built. Social learning is also widespread in the animal kingdom, but other animals have little capacity for cultural learning – the kind of social learning that supports cultural selection. Consequently, one would not expect to find cumulative cultural evolution of teaching outside the hominin line, or the associated ability to teach a broad range of skills. However, the teaching that does occur in non-human animals – for example, of hunting by domestic cats (see Hoppitt et al., 2008) – may be based on some of the same cognitive components as human teaching. This perspective suggests that the distinctiveness of human teaching depends on the extent to which its development involves integration of cognitive components. If cultural learning produces a new cognitive system for teaching – if it converts wheels, poles and wires into a cognitive bicycle – human teaching is likely to be highly distinctive in the animal kingdom. However, if cultural evolution leaves the old parts in a loose assembly, human teaching may differ quantitatively but not qualitatively from teaching in other animals.

Finally on future directions, it would be valuable to develop and test alternative accounts of the evolution of teaching that are intermediate between the instinct and gadget hypotheses discussed in this article. It is possible that the genetically inherited components of teaching are more diverse and less domain-specific than suggested by the natural cognition account, and more domain- and species-specific than suggested by the cognitive gadget account. This is implied, but not explicitly stated, in theories proposing that the development of teaching depends on human-specific, ‘universal’, and ‘early developing’ ‘psychological adaptations’ – such as ‘cognitive flexibility’, ‘prosociality’, ‘conformist bias’ and ‘prestige bias’ – that contribute not only to teaching, but also to other forms of ‘cumulative cultural learning’ (Kline, 2015; Legare, 2017, 2019; Lew-Levy et al., 2022). Human psychological characteristics can be universal, early developing and adaptive without being genetically inherited (Heyes, in press), but it is likely that these theories represent a kind of ‘weak nativism’ in relation to teaching. If so, to test them against the instinct and gadget accounts discussed in this article, it will be necessary to define the key components more clearly (e.g. what ‘cognitive flexibility’ is; Heyes & Moore, in press), to assess their contributions to teaching across cultures and, for each component, to scrutinise the evidence that it is genetically rather than culturally inherited. This work has begun for prosociality (Heyes, 2019b, in press), natural pedagogy (Heyes, 2016a) and social learning strategies such as conformist and prestige bias (Heyes, 2016b, c).

## 5. Conclusion

In this *Perspective* we have argued that human teaching is a ‘cognitive gadget’ (Heyes, 2018, 2019a) and offered a cultural evolutionary account suggesting that teaching is culturally transmitted. Ethnographic fieldwork and cross-cultural experimental studies show not only that adult-level teaching practices exhibit a high level of cross-cultural diversity, but also that being exposed to new teaching methods (for example through formal schooling) can change the way we teach. They further suggest that children from different cultural environments differ in their developmental trajectory of teaching, as the socialisation practices and value systems to which they are exposed influence both how they teach and how they think about teaching. Moreover, the ethnographic and historical record suggests that human teaching is subject to cumulative cultural evolution. We have further argued that our ability to acquire and implement teaching is rooted in a starter kit consisting of a range of genetically inherited traits (such as a high level of social tolerance, prosocial motivations and attentional biases, expanded associative learning abilities, and executive control). However, these are not specific to teaching; we argue that teaching is not a cognitive instinct. Instead, we have proposed that children learn to teach through a synthetic, plastic ontogenetic process by participating in teaching interactions with adults and peers, combining multiple mechanisms of cultural transmission (such as observation, imitation and evaluative feedback). This process hijacks ‘old parts’ – psychological mechanisms involved in prosocial conduct more broadly (such as those supporting bonding, coordination and communication) and a range of domain-general abilities (such as reinforcement learning and executive function) – rather than a suite of cognitive adaptations specifically for teaching. Experimental research suggests that these domain-general mechanisms are at least as important for human teaching as mindreading. While human teaching also involves some ‘new parts’ (such as conceptual communication through language), we have reason to believe that their cognitive underpinnings did not evolve specifically for teaching. We hope that debate on the evolution of teaching will inspire more empirical research on the cognitive basis and ontogeny of teaching across cultures.

## Data Availability

This article is conceptual in nature and does not rely on any primary data or code.
